# Teacher’s Type D Personality and Chinese Children’s Hyperactive Behaviors: Moderation Effect of Parental Type D Personality and Mediation Effect of Teacher–Student Relationship

**DOI:** 10.3389/fpsyg.2019.02517

**Published:** 2019-11-08

**Authors:** Guan-Hao He, Esben Strodl, Li Liu, Zeng-Liang Ruan, Xiao-Na Yin, Guo-Ming Wen, Deng-Li Sun, Dan-Xia Xian, Hui Jiang, Jin Jing, Yu Jin, Chuan-An Wu, Wei-Qing Chen

**Affiliations:** ^1^Department of Biostatistics and Epidemiology, School of Public Health, Sun Yat-sen University, Guangzhou, China; ^2^School of Psychology and Counselling, Queensland University of Technology, Brisbane, QLD, Australia; ^3^Women’s and Children’s Hospital of Longhua District, Shenzhen, China; ^4^Department of Women and Child Health, School of Public Health, Sun Yat-sen University, Guangzhou, China; ^5^Department of Information Management, Xinhua College of Sun Yat-sen University, Guangzhou, China

**Keywords:** hyperactive behaviors, type D personality, parent, teacher, children

## Abstract

This study aimed to explore the association between teacher’s type D personality (TDP) and children’s hyperactive behaviors, along with the moderation effect of parental TDP and the mediation effect of the teacher–student relationship. In this prospective study, a total of 25,852 children were surveyed from 2014 to 2016 in Longhua District of Shenzhen, China, and followed up 1 year later. At baseline, parents provided data on parental TDP and children’s hyperactive behaviors, while teachers reported on their TDP. At follow-up, parents provided data on children’s hyperactive behaviors again, and teachers described their relationship with each student. Two-level multilevel logistic models were conducted to assess the influence of a teacher’s TDP, parental TDP, and their interaction on children’s hyperactive behaviors. Mediation analysis was used to examine the mediating role of the teacher–student relationship. Results indicated that teachers’ TDP was not a significant predictor of children’s hyperactive behaviors after 1 year in kindergarten. Conversely, maternal and paternal TDP were prospectively and positively associated with children’s subsequent hyperactive behaviors. However, the children with a TDP teacher, a TDP mother, and/or a TDP father had higher risk of hyperactive behaviors than those with either a TDP teacher or a TDP mother or a TDP father. In addition, the teacher–student relationship was not a significant mediator between teacher’s TDP and children’s hyperactive behaviors. Further, researchers may consider the effect of the combination of teacher’s TDP, maternal TDP, and paternal TDP on hyperactive behaviors in children in further studies.

## Introduction

Hyperactive behavior is the main clinical manifestation of attention-deficit/hyperactivity disorder (ADHD) (American Psychiatric Association, 2013) and is one of the most common neurobehavioral conditions in early childhood (Gillberg, 2010). Children with hyperactive behaviors tend to encounter increased risk of adverse life problems such as poor academic performance (Taylor et al., 2019), employment difficulties and low income (Fletcher, 2014), injuries (Hurtig et al., 2016), and even suicide (Huang et al., 2018). In addition, preschool hyperactive behaviors also exert long-term economic burden upon families and country (Chorozoglou et al., 2015). Hyperactive behaviors can emerge in early childhood and continue into adulthood, which may lead to a lifetime dysfunction without effective treatment or prevention (Barkley, 2014). Therefore, there is a pressing need to explore the risk factors for hyperactive behaviors in young children in order to develop effective prevention and intervention programs.

Although genetic and biological factors are central to the etiology of hyperactive behaviors (Thapar et al., 2012), empirical studies indicate that environmental factors including families and schools can play an important role in determining the development of hyperactive behaviors (Keown and Woodward, 2002; Keown, 2012; Runions, 2014). In terms of the familial factors, parental personality, which refers to their relatively enduring styles of thinking, feeling, and acting, can have an initial influence on the parental rearing behaviors and children’s hyperactive behaviors. For example, Prinzie et al. demonstrated that parents with the personality dimension of low emotional stability tended to exhibit more parenting behaviors of irritability, anger, meanness, and frustration, which was subsequently associated with an increase in symptoms of externalizing behavior problem (including hyperactive behaviors) in their children (Prinzie et al., 2004). In addition, a meta-analysis of Chinese studies indicated that the maternal personality of extraversion was protective against the subsequent development of hyperactive behaviors in their children (Zhang and Sun, 2015). Furthermore, our previous research found that parental type D personality (TDP) was a risk factor for childhood hyperactive behaviors (He et al., 2019). These findings suggested that the caregivers’ personality can make a contribution to the hyperactive behaviors of their offspring.

In addition to parents, teachers are important caregivers of children after they enter kindergartens and schools. Children’s early experiences with teachers have been reported to significantly affect the development of children’s hyperactive behaviors. The results of a meta-analysis demonstrated that there was a negative correlation between positive teacher–student relationships and students externalizing behavioral problems (Lei et al., 2016). More specially, a prospective study by Runions et al. reported that conflict between a teacher and children in kindergarten could predict a subsequent increase in children’s hyperactivity (Runions, 2014). Further, Searle et al. (2013) demonstrated that good teacher–student relationship quality was associated with lower levels of children’s hyperactivity. In addition, an RCT study showed that intervention targeted at teacher–student relationship could reduce hyperactive behaviors among preschoolers (Vancraeyveldt et al., 2015). However, scarce attention has been paid to the influence of teachers’ personality, which may be a driver of their interaction with students, and in turn affect their students’ hyperactive behaviors. For instance, Eryilmaz (2014) reported that teachers with a personality of high extraversion, agreeableness, conscientious, openness personality, and low neuroticism were more popular with students. Similarly, Genc et al. (2014) also found that students think that a good teacher is expected to have personality of low neuroticism and high extraversion, openness, cooperativeness, and consciousness. A literature review indicated that a teacher’s ability to teach children with behavioral problems (including building a good teacher–student relationships) increased with his/her levels in personality of extraversion, agreeableness, conscientious, and openness (Buttner et al., 2015). Given that previous research has found a link between teachers’ personality and teacher–student relationships, as well as an association between teacher–student relationships and levels of hyperactivity in students, it is likely that there may be a relationship between a teacher’s personality and a student’s level of hyperactive behaviors.

Type D personality is a personality construct characteristic of a combination of two traits: negative affectivity (NA) and social inhibition (SI) (Denollet, 2005). NA refers to the propensity to experience negative emotions such as anxiety, dysphoria, and irritability and involves a negative view of self. SI denotes suffering discomfort in social contacts, reticence, lack of social interaction, and unwillingness to express in social poise. There is evidence to show that TDP is associated with fewer healthy behaviors, poorer physical status, as well as a poorer mental health status (Mols and Denollet, 2010; Williams et al., 2016). Particularly, Thomas et al. (2006) demonstrated that teachers with TDP tended to be bothered more by voice problems than their non-TDP counterparts. Although few researches have paid attention to the association between teacher’s TDP and children’s hyperactive behaviors, our previous study found that TDP parents tend to have fewer positive parent–child interactions, which, in turn, increases the risk of children’s hyperactive behaviors (He et al., 2019). However, there was limited evidence on the association between teacher’s TDP (another important caregiver) and children’s hyperactive behaviors. Based on the findings of previous studies, we hypothesized that teachers with TDP may also establish negative relationships with their students, which may subsequently influence children’s hyperactive behaviors. In addition to the independent effect of teacher’s TDP, it is also noteworthy that teacher’s TDP and parents’ TDP may combine together to influence children’s hyperactive behaviors after kindergarten. However, to date, there have been no published investigations into this issue. Examining whether such associations exist may be important in understanding the development of hyperactive behaviors in children.

The present study therefore aimed to explore the influence of teacher’s TDP on the development of hyperactive behaviors in children, as well as the mediating role of teacher–student relationship. In addition, we also aimed to explore the interaction effect among maternal TDP, paternal TDP, and teacher’s TDP. Given these aims, the hypotheses of the present study are as follows:

(1) Teacher’s TDP will be associated with children’s subsequent hyperactive behaviors after a 1-year period in kindergarten.
(2) There will be a significant interaction effect between maternal TDP, paternal TDP, and teachers’ TDP with children’s subsequent hyperactive behaviors after a 1-year-period in kindergarten.
(3) Three types of teacher–student relationships (conflict, closeness, and dependency) will mediate the relationship between teacher TDP and the children’s subsequent hyperactive behaviors.

## Materials and Methods

### Study Population

**FIGURE 1:**
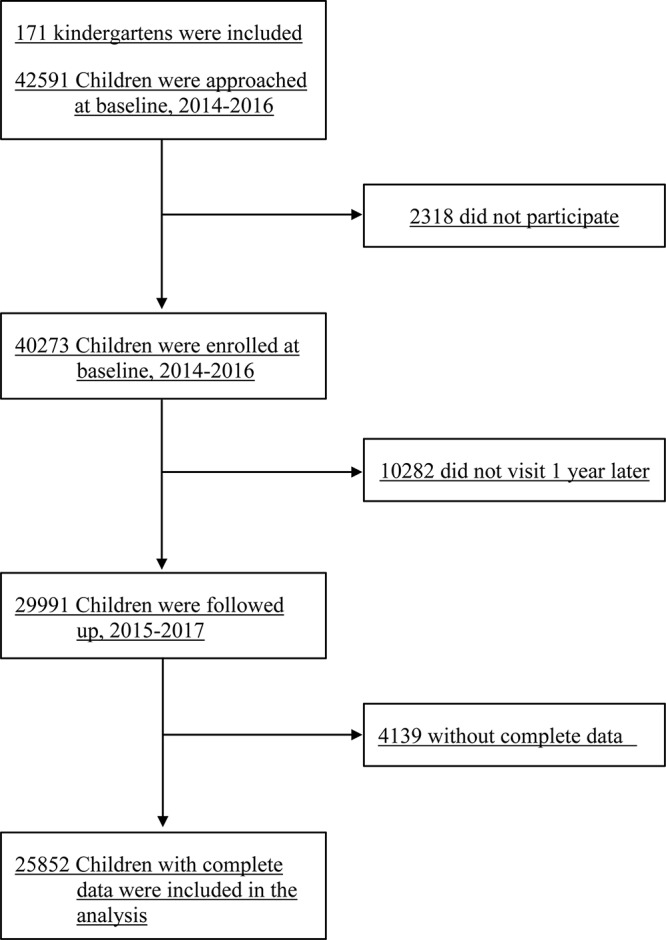
Flowchart of study participants.

The Longhua Children Cohort Study (LCCS) was set up to estimate the influence of early-life family and school environment upon psycho-behavioral development of Chinese children. The baseline survey was conducted from 2014 to 2016. Children and their parents and teachers in the 171 kindergartens in the Longhua district of Shenzhen, China, were invited to participate in this study voluntarily when they first entered kindergarten. The exclusion criteria were children with severe physical illness or mental disorder. A total of 42,591 children aged around 3 years were approached at baseline survey, and 40,273 children were enrolled in the study, with a participation rate of 94.56%. In addition, a total of 1234 teachers of the children were also included in the current study. The follow-up survey was conducted 1 year later and a total of 29,991 children were successfully recruited at follow-up. After excluding those with incomplete data, the remaining 25,852 children were included in the analyses (Figure 1). Written informed consent was provided by the children’s parents and teachers. The current study was conducted in accordance with the Code of Ethics of the Word Medical Association (Declaration of Helsinki). The current study was approved by the Ethics Committee of School of Public Health of Sun Yat-sen University (ethics clearance No. 2015-016).

### Data Collection

At baseline, both the mother and father of a child were required to fill in a scale about TDP, and one of the parents was also asked to complete an additional questionnaire concerning the socio-demographics of their children and both parents, as well as answering questions about the hyperactive behaviors of their child entering kindergarten. Teachers were also asked to complete a scale about TDP and their socio-demographics at this baseline time point. At follow-up, the parents were asked to complete another scale about their child’s hyperactive behaviors. In order to explore how teacher’s TDP influence children’s hyperactive behaviors, a subsample of teachers was also asked to fill in a scale regarding their relationship with each child. That is, teachers of children who participated in this study in 2014 provided information about their relationship with each child in their class at 2015 (*n* = 6387).

### Measures

#### Hyperactive Behaviors

Children’s hyperactive behaviors were assessed using the Conners’ hyperactivity index (HI), which is a subscale from the Conners Parent Rating Scale-Revised (CPRS-48) (Goyette et al., 1978). The parent version of Conners’ HI is a validated screening tool, which has been widely used in epidemiological studies to measure hyperactive behaviors in preschool children (Heinonen et al., 2010; Zhu et al., 2015). This scale was translated into Chinese and proven to have a good reliability and validity (Lin-Yan et al., 2001). In our sample, the Cronbach α coefficients was 0.83, the composite reliability was 0.94 and the average variance extracted (AVE) was 0.77. The results of a confirmatory factor analysis (CFA) revealed that HI had good construct validity (most of items had factor loadings above 0.60, CFI = 0.91, GFI = 0.95, RMSEA = 0.08). These findings indicated that the HI is a reliable and validated tool for the measurement of Chinese children’s hyperactive behaviors. The HI consists of 10 items rated on a four-point scale (0 = not at all to 3 = very much). The items were summed and then divided by 10 to get a mean score. The 90th percent HI score for the child’s age and gender is used as the cutoff for establishing hyperactive behaviors in Chinese children (Lin-Yan et al., 2001).

#### Type D Personality

Type D personality scale (DS14) was used to assess TDP of parents and teachers. The DS14 is composed of two factors, NA and SI. Each factor involved seven items rated on a five-point scale (0 = false to 4 = true). The score ranges from 0 to 28 for both NA and SI. Respondents with a score ≥ 10 on both subscales are regarded as having a TDP (Denollet, 2005). The DS14 was translated into Chinese and demonstrated to have a good reliability and validity (Bai, 2007). The Chinese version of DS14 has been extensively used to evaluate TDP among Chinese sample in epidemiological and clinical studies (Chen et al., 2015; Shao et al., 2017). In our sample, the Cronbach α coefficients of NA were 0.90 for mother, 0.87 for father, and 0.82 for teacher, and the Cronbach α coefficients of SI were 0.70 for mother, 0.73 for father, and 0.69 for teacher. The composite reliability of NA was 0.92 for mother, 0.89 for father, and 0.85 for teacher, and the composite reliability of SI was 0.73 for mother, 0.75 for father, and 0.73 for teacher. The AVE of NA was 0.80 for mother, 0.85 for father, and 0.78 for teacher, and the AVE of SI was 0.67 for mother, 0.66 for father, and 0.78 for teacher. According to the Fornell and Larcker (1981) criterion, the two factors of DS14 had good discriminant validity. The AVE of NA were all larger than the squared correlation coefficient between NA and SI (for mother: 0.80 > 0.62, for father: 0.85 > 0.62, and for teacher: 0.78 > 0.52). Similarly, the AVE of SI were all larger than the squared correlation coefficient between NA and SI (for mother: 0.67 > 0.62, for father: 0.66 > 0.62, and for teacher: 0.78 > 0.52). The construct validity of DS14 (two factor model) is acceptable (most of items had factor loadings above 0.60, for mother: CFI = 0.91, GFI = 0.90, RMSEA = 0.09; for father: CFI = 0.90, GFI = 0.91, RMSEA = 0.09; and for teacher: CFI = 0.90, GFI = 0.90, RMSEA = 0.09). These findings demonstrated that the DS14 is a reliable and validated tool to measure TDP among Chinese population.

#### Teacher–Student Relationship

The teacher–student relationship was measured using the student–teacher relationship scale (STRS) (Pianta, 2001). This scale is composed of three subscales: Conflict, Closeness, and Dependency. The subscale of Conflict measures the degree to which a teacher perceives his or her relationship with a particular student as negative and conflictual. The subscale of Closeness measures the degree to which a teacher experiences affection, warmth, and open communication with a particular student. The subscale of Dependency measures the degree to which a teacher perceives a particular student as overly dependent on him/her. Researchers have translated this scale into Chinese and proved to have a stratifying reliability and validity among preschoolers (Zhang, 2010). The Chinese version STRS has been employed to evaluate teacher–student relationships in a series of psychological and educational study in Chinese sample (Lizhu et al., 2016; Jingjing et al., 2018). In our sample, the Cronbach α coefficient was 0.85 for Conflict, 0.78 for Closeness, and 0.56 for Dependency, and the composite reliability was 0.87 for Conflict, 0.78 for Closeness, and 0.51 for Dependency. The AVE was 0.66 for Conflict, 0.60 for Closeness, and 0.43 for Dependency. In addition, the STRS also had acceptable discriminant validity. The AVE of Conflict was larger than the squared correlation coefficients between Conflict and the other two subscales (0.66 > 0.10, 0.66 > 0.37). The AVE of Closeness was larger than the squared correlation coefficients between Closeness and the other two subscales (0.60 > 0.10, 0.60 > 0.06). The AVE of dependency was larger than the squared correlation coefficients between Dependency and the other two subscales (0.43 > 0.37, 0.60 > 0.06). The STRS (three-factor model) also had good construct validity (most of the items had factor loadings above 0.50, CFI = 0.90, GFI = 0.90, and RMSEA = 0.06).

#### Covariates

The potential covariate variables included parents’ and teachers’ sociodemographic characteristics of age, educational level (≤12th grade, high school, and undergraduate), the parent’s marital status (married and single), the teacher’s gender (male and female), as well as children’s sociodemographic characteristics of age, gender (male and female), and single child status (no and yes). The variables that were significant at *P* < 0.1 in univariate analyses or widely reported in the literatures were entered as covariates into the multiple regression models.

### Statistical Analysis

Continuous variables were described as means and standardized deviation, while categorical variables were described as frequencies and proportion. *t* tests were performed for the comparisons for continuous variables, and χ^2^ tests were used for the comparisons for categorical variables. Our data had class-level variables (including teacher’s age, teacher’s gender, teacher’s educational level, and teacher’s TDP) and child-level variables (including the child’s age, gender, single child status, as well as parents’ age, parents’ educational level, parents’ marital status, and parental TDP). As such, we built two-level logistic models to investigate the associations between teacher’s TDP and children’s hyperactive behaviors after adjusting for child-level data (level-1) and class-level data (level-2) (O’Connell et al., 2008).

The potential for the teacher–student relationship to mediate the association between teacher’s TDP and children’s hyperactive behaviors was assessed with a series of hierarchical logistic regressions after adjusting for the covariate variables. According to Baron and Kenny (1986), the mediation effect is demonstrated when the following criteria are met: (1) The main independent variable (teacher’s TDP) is significantly related to the main dependent variable (hyperactive behaviors of children, the partial coefficient was denoted by c); (2) The independent variable (teacher’s TDP) is significantly associated with the mediator variable (teacher–student relationship, the partial coefficient was denoted by a); and (3) The mediator variable (teacher–student relationship) is significantly related to the dependent variable (hyperactive behaviors of children) when the independent variable (teacher’s TDP) is controlled (the partial coefficient was denoted by b).

All of the *p* values were two-sided. Type I errors were set at *P* < 0.05. The statistical analysis was conducted with SAS 9.4 (SAS Institute Inc., Cary, NC, United States).

## Results

### Sample Characteristics

Of the surveyed 29,991 children, 54.5% were boys and the mean age was 3.43 years (*SD* = 0.35). Further, 56.0% of mothers and 63.9% of fathers had a bachelor degree or above. The mean age was 30.91 years (*SD* = 3.92) for mothers and 33.27 years (*SD* = 4.55) for fathers. In addition, 60.4% of teachers had a bachelor degree or above, and their mean age was 26.16 years (*SD* = 6.53). Other socio-demographic characteristics of participants are shown in Table 1.

**TABLE 1:** Characteristics of children with and without hyperactive behaviors at follow-up.

**Variables**	**Total**	**Hyperactive behaviors at follow-up**		
		
		**No**	**Yes**	***P* value^a^**
Total, *n* (%)	25,852(100.0)	23,905(92.47)	1947 (7.53)	–
Child age at baseline, mean (SD)	3.43 (0.35)	3.44 (0.35)	3.38 (0.36)	<0.001
Child’s gender, *n* (%)				0.496
Boy	14,093(54.51)	13,046(54.57)	1047 (53.78)	
Girl	11,759(45.49)	10,859(45.43)	900 (46.22)	
Single child, *n* (%)				0.013
No	10,527(40.72)	9786 (40.94)	741 (38.06)	
Yes	15,325(59.28)	14,119(59.06)	1206 (61.94)	
Marital status, *n* (%)				0.724
Married	25,141(97.25)	23,250(97.26)	1891 (97.12)	
Single	711 (2.75)	655 (2.74)	56 (2.88)	
Maternal age, mean (SD)	30.91 (3.92)	31.00 (3.92)	29.76 (3.83)	<0.001
Maternal education, *n* (%)				<0.001
≤12th grade	3939 (15.24)	3505 (14.66)	434 (22.29)	
≤High school	7457 (28.84)	6849 (28.65)	608 (31.23)	
≥Undergraduate	14,456(55.92)	13,551(56.69)	905 (46.48)	
Paternal age, mean (SD)	33.27 (4.55)	33.35 (4.55)	32.24 (4.52)	<0.001
Paternal education, *n* (%)				<0.001
≤12th grade	3073 (11.89)	2730 (11.42)	343 (17.62)	
≤High school	6255 (24.20)	5733 (23.98)	522 (26.81)	
≥Undergraduate	16,524(63.92)	15,442(64.60)	1082 (55.57)	
Teacher’s gender, *n* (%)				0.291
Male	136 (0.53)	129 (0.54)	7 (0.36)	
Female	25,716(99.47)	23,776(99.46)	1940 (99.64)	
Teacher’s age, mean (SD)	26.12 (6.53)	26.16 (6.54)	25.57 (6.32)	<0.001
Teacher’s education, *n* (%)				0.001
≤12th grade	502 (1.94)	459 (1.92)	43 (2.21)	
≤High school	9734 (37.65)	8930 (37.36)	804 (41.29)	
≥Undergraduate	15,616(60.41)	14,516(60.72)	1100 (56.50)	
Hyperactive behaviors at baseline *n* (%)				<0.001
No	21,274(82.29)	20,337(85.07)	937 (48.13)	
Yes	4578 (17.71)	3568 (14.93)	1010 (51.87)	

*^*a*^χ2 tests were used for categorical variables; *t* tests were used for continuous variables.*

Based on the recommended cutoff value for the DS14, 11.1% of mothers, 9.1% of fathers, and 9.8% of teachers were classified as TDP. Regarding the trajectory of the children’s hyperactive behaviors, there were 935 out of the 21,231 baseline non-hyperactive children who subsequently met the cutoff value of hyperactive behaviors at follow-up. A total of 3565 out of the 4574 baseline hyperactive children subsequently were rated lower than cutoff for hyperactivity at follow-up and so considered to be in remission.

### Association Between Teacher’s TDP and Children’s Hyperactive Behaviors, and the Moderation Effect of Parental TDP

Table 2 presents the results of the associations of baseline teacher’s TDP and parental TDP with children’s hyperactive behaviors at follow-up. After adjusting for the covariates, teacher’s TDP at baseline did not predict the children’s hyperactive behaviors after 1 year in kindergarten independently. In contrast, maternal TDP and paternal TDP at baseline were independent and positive predictors of children’s hyperactive behaviors at follow-up. Further, we explored the moderation effect of parental TDP on the association between teacher’s TDP and children’s hyperactive behaviors, but no significant moderation effect of parental TDP was observed (Table 2).

**TABLE 2:** Association of parental TDP and teacher’s TDP with hyperactive behaviors at follow-up among children with and without hyperactive behaviors at baseline.

**Variable**	**Crude OR (95% CI)**	**Adjusted OR (95% CI)**
**Outcome: Hyperactive behaviors (*n* = 25,852)**		
Teacher’s TDP (Yes vs. No)	1.051(0.885,1.249)	1.048(0.883,1.243)
Maternal TDP (Yes vs. No)	3.207(2.864,3.591)^***^	2.413(2.138,2.724)^***^
Paternal TDP (Yes vs. No)	2.641(2.330,2.993)^***^	2.140(1.871,2.447)^***^
**Interaction effect**		
Teacher’s TDP^∗^Maternal TDP^∗^Paternal TDP	0.691(0.409,1.169)	0.709(0.406,1.237)
Teacher’s TDP^∗^Maternal TDP	1.235(0.859,1.776)	1.365(0.926,2.013)
Teacher’s TDP^∗^Paternal TDP	0.859(0.559,1.322)	0.843(0.534,1.332)
Maternal TDP^∗^Paternal TDP	0.704(0.533,0.929)^∗^	0.854(0.634,1.150)
**Outcome: Incidence of hyperactive behaviors (*n* = 21,274)**		
Teacher’s TDP (Yes vs. No)	1.005(0.791,1.276)	0.992(0.786,1.253)
Maternal TDP (Yes vs. No)	3.150(2.678,3.705)^***^	3.107(2.641,3.656)^***^
Paternal TDP (Yes vs. No)	2.484(2.037,2.978)^***^	2.528(2.108,3.031)^***^
**Interaction effect**		
Teacher’s TDP^∗^Maternal TDP^∗^Paternal TDP	0.435(0.178,1.062)	0.423(0.172,1.045)
Teacher’s TDP^∗^Maternal TDP	1.030(0.606,1.751)	0.993(0.578,1.707)
Teacher’s TDP^∗^Paternal TDP	0.625(0.315,1.241)	0.610(0.304,1.224)
Maternal TDP^∗^Paternal TDP	0.848(0.564,1.227)	0.919(0.606,1.396)
**Outcome: Persistence of hyperactive behaviors (*n* = 4578)**		
Teacher’s TDP (Yes vs. No)	1.174(0.918,1.501)	1.128(0.882,1.443)
Maternal TDP (Yes vs. No)	2.156(1.818,2.556)^***^	2.133(1.797,2.531)^***^
Paternal TDP (Yes vs. No)	2.036(1.684,2.461)^***^	2.058(1.699,2.493)^***^
**Interaction effect**		
Teacher’s TDP^∗^Maternal TDP^∗^Paternal TDP	0.972(0.458,2.064)	1.075(0.497,2.324)
Teacher’s TDP^∗^Maternal TDP	1.797(1.015,3.182)^∗^	1.962(1.090,3.534)^∗^
Teacher’s TDP^∗^Paternal TDP	1.059(0.564,1.989)	1.113(0.583,2.127)
Maternal TDP^∗^Paternal TDP	0.782(0.518,1.181)	0.791(0.517,1.211)

*^∗^*P* < 0.05 and ^∗∗∗^*P* < 0.001. Adjusted for child gender, child age, single child, child’s baseline hyperactive index, marital status, maternal age, maternal education, paternal age, paternal education, teacher’s age, teacher’s gender, and teacher’s education.*

To explore the influence of teacher’s TDP and parental TDP with the change of the status of children’s hyperactive behaviors after a year’s life in kindergarten, we classified the children into two subgroups at baseline: non-hyperactive behaviors children and hyperactive behaviors children, based on the HI at baseline survey. For the children who did not exhibit hyperactive behaviors at baseline, their hyperactive behaviors status (1 = yes and 0 = no) at follow-up was used as dependent variable. There was no significant association between teacher’s TDP and the incident of children’s hyperactive behaviors. In contrast, both maternal TDP and paternal TDP were significantly and positively associated with higher risk of incident of children’s hyperactive behaviors before and after adjusting for the covariates (Table 2). Moreover, there was no moderation effect of parental TDP on the association between teacher’s TDP to the incident of children’s hyperactive behaviors (Table 2).

For the children who exhibited hyperactive behaviors at baseline, children’s hyperactive behaviors status at follow-up was used as dependent variable (1 = persistence and 0 = remission). No association between teacher’s TDP and the persistence of children’s hyperactive behaviors was observed. Conversely, both maternal TDP and paternal TDP were significantly and positively associated with the persistence of children’s hyperactive behaviors before and after adjusting for the covariates. Additionally, the moderation effect of maternal TDP on the relationship between teacher’s TDP and the persistence of children’s hyperactive behaviors was positively significant (Table 2).

### Association of the Combination of Teacher’s TDP, Maternal TDP, and Paternal TDP With Children’s Hyperactive Behaviors

In order to explore the influence of teacher’s TDP, maternal TDP, and paternal TDP concurrently, these three variables were combined into an extra variable. This variable included eight combinations of the presence or absence of teacher’s, maternal, and paternal TDP to produce eight categories. Table 3 presented the association of the combination of maternal TDP, paternal TDP, and teacher’s TDP on children’s hyperactive behaviors. For the total sample, compared to children with a non-TDP mother, non-TDP father, and non-TDP teacher, those just having a parent with TDP, those having both TDP father and TDP mother (teacher was not TDP), those having a TDP mother and a TDP teacher (father was not TDP), and those having a TDP mother, a TDP father, and a TDP teacher had higher risk of subsequent hyperactive behaviors. The latter three seemed to have higher absolute OR. For the children who did not exhibit hyperactive behaviors at baseline, those with a TDP father and a TDP mother (teacher was not TDP) and those with a TDP mother and a TDP teacher (father was not TDP) encountered higher risk of subsequent incidence of hyperactive behaviors. The latter two also had higher absolute OR. For the children who exhibited hyperactive behaviors at baseline, the results were similar with the total sample (Table 3).

**TABLE 3:** Association of maternal TDP, paternal TDP, and teacher’s TDP with children’s hyperactive behaviors at follow-up.

**Variable**			**No.**	**Case**	**Crude OR (95% CI)**	**Adjusted OR (95% CI)**
**Outcome: Hyperactive behaviors (*n* = 25,852)**						
**Maternal TDP**	**Paternal TDP**	**Teacher’s TDP**				
No	No	No	19,666	1190	Ref	Ref
Yes	No	No	1522	224	2.743(2.346,3.208)^***^	1.994(1.686,2.358)^***^
No	Yes	No	1062	118	1.979(1.615,2.452)^***^	1.637(1.319,2.031)^***^
No	No	Yes	2154	130	1.000(0.814,1.230)	0.973(0.792,1.196)
Yes	Yes	No	1061	216	4.137(3.507,4.880)^***^	3.121(2.612,3.728)^***^
Yes	No	Yes	164	36	4.532(3.061,6.710)^***^	3.904(2.565,5.942)^***^
No	Yes	Yes	105	12	2.084(1.120,3.875)^∗^	1.708(0.887,3.290)
Yes	Yes	Yes	118	21	3.432(2.095,2.623)^***^	2.446(1.454,4.117)^***^
**Outcome: Incidence of hyperactive behaviors (*n* = 21,274)**						
**Maternal TDP**	**Paternal TDP**	**Teacher’s TDP**				
No	No	No	16,516	600	Ref	Ref
Yes	No	No	1083	95	2.607(2.072,3.279)^***^	2.612(2.076,3.286)^***^
No	Yes	No	813	48	1.680(1.237,2.282)^***^	1.762(1.297,2.394)^∗∗^
No	No	Yes	1822	66	0.988(0.747,1.307)	0.980(0.744,1.290)
Yes	Yes	No	749	100	4.212(3.342,5.310)^***^	4.230(3.354,5.334)^***^
Yes	No	Yes	127	17	4.244(2.479,7.267)^***^	4.409(2.574,7.551)^***^
No	Yes	Yes	82	5	1.769(0.701,4.464)	1.817(0.716,4.610)
Yes	Yes	Yes	82	6	2.012(0.856,4.732)	1.918(0.819,4.494)
**Outcome: Persistence of hyperactive behaviors (*n* = 4578)**						
**Maternal TDP**	**Paternal TDP**	**Teacher’s TDP**				
No	No	No	3150	590	Ref	Ref
Yes	No	No	439	129	1.825(1.451,2.297)^***^	1.832(1.454,2.308)^***^
No	Yes	No	249	70	1.731(1.285,2.330)^***^	1.791(1.327,2.417)^***^
No	No	Yes	332	64	1.037(0.767,1.402)	0.980(0.723,1.327)
Yes	Yes	No	312	116	2.643(2.049,3.410)^***^	2.617(2.024,3.384)^***^
Yes	No	Yes	37	19	4.796(2.443,9.414)^***^	4.646(2.352,9.177)^***^
No	Yes	Yes	23	7	1.956(0.780,4.905)	1.808(0.715,4.571)
Yes	Yes	Yes	36	15	3.316(1.658,6.633)^***^	3.119(1.550,6.278)^∗∗^

*^∗^*P* < 0.05, ^∗∗^*P* < 0.01, and ^∗∗∗^*P* < 0.001. Adjusted for child gender, child age, single child, child’s baseline hyperactive index, marital status, maternal age, maternal education, paternal age, paternal education, teacher’s age, teacher’s gender, and teacher’s education.*

### The Mediating Role of Teacher–Student Relationship Between Teachers’ TDP and Children’s Hyperactive Behaviors

**FIGURE 2:**
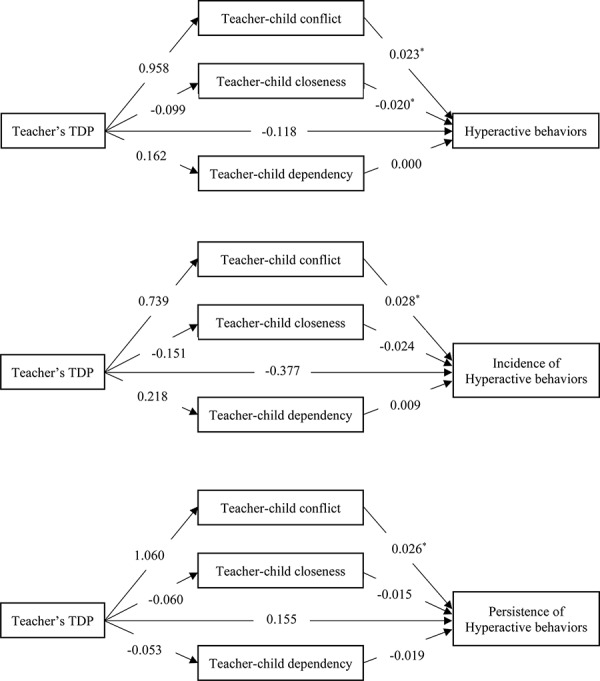
The mediating role of teacher–children relation on the association between teacher’s TDP and children’s. ^∗^*P* < 0.05.

Table 4 and Figure 2 showed the results of the mediation analysis of teacher–student relationship. The results indicated that the teacher’s TDP was not associated with teacher–student relationship as well as children’s hyperactive behaviors, though the teacher–student conflict was related to higher risk of children’s hyperactive behaviors, while teacher–student closeness was associated with lower risk of children’s hyperactive behaviors. Therefore, the teacher–student relationships did not serve as significant mediators.

**TABLE 4:** The mediating role of teacher–children relation on the association between teacher’s TDP and children’ hyperactive behaviors.

	**Independent variable**	**Dependent variable**	**Effect value (95% CI)**
**Outcome: Hyperactive behaviors (*n* = 6387)**			
X→Y	Teacher’s TDP	Hyperactive behaviors	−0.092(−0.467,0.283)
X→M	Teacher’s TDP	Teacher-child conflict	0.958(−0.650,2.566)
X→M	Teacher’s TDP	Teacher-child closeness	−0.099(−1.522,1.323)
X→M	Teacher’s TDP	Teacher-child dependency	0.162(−0.651,0.974)
M→Y	Teacher–child conflict	Hyperactive behaviors	0.028(0.014,0.041)^***^
M→Y	Teacher–child closeness	Hyperactive behaviors	−0.028(−0.043,−0.012)^***^
M→Y	Teacher–child dependency	Hyperactive behaviors	0.010(−0.017,0.036)
X + M→Y	Teacher’s TDP	Hyperactive behaviors	−0.118(−0.494,0.259)
	Teacher–child conflict	Hyperactive behaviors	0.023(0.006,0.039)^∗^
	Teacher–child closeness	Hyperactive behaviors	−0.020(−0.038,−0.001)^∗^
	Teacher–child dependency	Hyperactive behaviors	0.000(−0.032,0.032)
**Outcome: Incidence of hyperactive behaviors (*n* = 4951)**			
X→Y	Teacher’s TDP	Hyperactive behaviors	−0.340(−0.919,0.239)
X→M	Teacher’s TDP	Teacher–child conflict	0.739(−0.861,2.338)
X→M	Teacher’s TDP	Teacher–child closeness	−0.151(−1.597,1.294)
X→M	Teacher’s TDP	Teacher–child dependency	0.218(−0.598,1.035)
M→Y	Teacher–child conflict	Hyperactive behaviors	0.035(0.017,0.053)^***^
M→Y	Teacher–child closeness	Hyperactive behaviors	−0.031(−0.052,−0.010)^∗∗^
M→Y	Teacher–child dependency	Hyperactive behaviors	0.021(−0.015,0.057)
X + M→Y	Teacher’s TDP	Hyperactive behaviors	−0.377(−0.951,0.197)
	Teacher–child conflict	Hyperactive behaviors	0.028(0.006,0.050)^∗^
	Teacher–child closeness	Hyperactive behaviors	−0.024(−0.048,0.001)
	Teacher–child dependency	Hyperactive behaviors	0.009(−0.034,0.053)
**Outcome: Persistence of hyperactive behaviors (*n* = 1436)**			
X→Y	Teacher’s TDP	Hyperactive behaviors	0.180(−0.304,0.664)
X→M	Teacher’s TDP	Teacher–child conflict	1.060(−0.867,2.987)
X→M	Teacher’s TDP	Teacher–child closeness	−0.060(−1.717,1.597)
X→M	Teacher’s TDP	Teacher–child dependency	−0.053(−1.062,0.955)
M→Y	Teacher–child conflict	Hyperactive behaviors	0.026(0.007,0.045)^∗∗^
M→Y	Teacher–child closeness	Hyperactive behaviors	−0.028(−0.051,−0.006)^∗^
M→Y	Teacher–child dependency	Hyperactive behaviors	−0.006(−0.043,0.030)
X + M→Y	Teacher’s TDP	Hyperactive behaviors	0.155(−0.338,0.648)
	Teacher–child conflict	Hyperactive behaviors	0.026(0.002,0.049)^∗^
	Teacher–child closeness	Hyperactive behaviors	−0.015(−0.042,0.012)
	Teacher–child dependency	Hyperactive behaviors	−0.019(−0.064,0.025)

*^∗^*P* < 0.05, ^∗∗^*P* < 0.01, and ^∗∗∗^*P* < 0.001. Adjusted for child gender, child age, single child, child’s baseline hyperactive index, marital status, maternal age, maternal education, paternal age, paternal education, teacher’s age, teacher’s gender, and teacher’s education.*

## Discussion

The current study explored the prospective association between teacher’s TDP and Chinese preschool children’s hyperactive behaviors over a 1-year period, as well as the moderation effect of parental TDP and the mediation effect of teacher–student relationships. After controlling for parent’s age, educational level, and marital status, and teacher’s age, gender, educational level, as well as children’s age, gender, and single child status, teachers’ TDP was not a significant predictor of children’s hyperactive behaviors after 1 year in kindergarten. Conversely, both maternal and paternal TDP at baseline were prospectively associated with higher risk of children’s subsequent hyperactive behaviors. After combining maternal TDP, paternal TDP as well as teacher’s TDP, we found that children having just one parent with TDP, those having both TDP father and TDP mother, those having a TDP mother and a TDP teacher, and those having a TDP mother, a TDP father, and a TDP teacher had higher risk of later hyperactive behaviors. Moreover, mediation analysis demonstrated that teacher–student relationships were not significant mediators between teacher’s TDP and children’s hyperactive behaviors, although teacher–student conflict was associated with higher risk of children’s hyperactive behaviors, while teacher–student closeness was related to lower risk of children’s hyperactive behaviors.

To the best of our knowledge, this is the first study that has explored a prospective association between teacher’s TDP and subsequent hyperactive behaviors among children. In contrast to our hypothesis, no association between teacher’s TDP and subsequent hyperactive behaviors among children was found in the current study. Given that previous studies have found associations between teachers’ personality and teacher’s popularity among students (Eryilmaz, 2014), as well as between teacher–student relationship quality and children’s hyperactivity (Searle et al., 2013), it would seem reasonable to predict a direct relationship between teacher’s personality and the presence of hyperactive behaviors in children. In our study, however, we did not observe any direct relationship between teacher’s TDP and children’s hyperactive behaviors.

A consideration for the non-significant finding between teacher TDP and hyperactivity is that the results may represent a dose effect. That is, while the children may spend a number of hours with a teacher each day, the amount of teacher–student interactions is mitigated by the higher number of competing children in the classroom, compared with the interactions between parent–child in a Chinese family. As such, having a teacher with TDP may not provide a sufficient dosage of exposure to a significant other with TDP in comparison with having a parent with TDP. Given this, teacher’s TDP when combined with parents’ TDP may increase the dosage of exposure and therefore influence children’s hyperactive behaviors.

While the lack of association between teacher TDP and hyperactive behaviors precluded a formal mediation analysis, our study still allowed an examination of the associations between teacher TDP, teacher–student relationships, and hyperactive behaviors in young Chinese children. We found that while there were no associations between teacher TDP and the three measures of teacher–student relationships, two types of teacher–student relationship (Conflict, Closeness) were related to hyperactive behaviors in Chinese children. This is similar to a finding from a prospective study conducted by Jong that teachers’ personality of extraversion and friendliness was not associated with later teacher–student relationship in secondary school in Netherlands (de Jong et al., 2014). However, other studies conversely demonstrated that students tended to like a teacher with personality of high extraversion, agreeableness, conscientious, openness personality, and low neuroticism (Eryilmaz, 2014; Genc et al., 2014). These contradictory findings may be attributed to different respondents (i.e., students vs. teachers) or different measuring methods of teacher–student relationships (i.e., qualitative questions vs. quantitative scale). Another consideration may be cultural influence. Teachers have high social status in Chinese Confucian culture. Chinese children are educated to be respectful to teachers since a very early age. There is cross-cultural evidence that Chinese students report better teacher–student relationships than American students (Yang et al., 2013). More relevant to our study, a previous study conducted among teachers of Belgian preschoolers reported that the average score is 3.87 for Conflict, 2.05 for Closeness, and 2.26 for Dependency (Doumen et al., 2009). In the current study, the average scores of STRS is numerically better, 1.64 for Conflict, 3.99 for Closeness, and 2.56 for Dependency. Given that, Chinese children may also establish a relatively good relationship with their teachers even though their teacher had TDP. Therefore, further studies may consider to examine the associations between teacher’s TDP and teacher–student relationships in other cultures, as well as to explore the relationships between teacher’s TDP and other specific variables of teacher–student interaction.

Though the association between teacher TDP and teacher–student relationships cannot be observed in this study, two types of teacher–student relationship (Conflict and Closeness) were found to be related to hyperactive behaviors in Chinese children. As indicated by Lei et al. (2016) teacher–student relationship played an important role in student’s behavioral development. Moreover, children with a conflict relationship with a teacher had higher levels of subsequent hyperactivity (Runions, 2014), while students with good teacher–student relationship quality had lower levels of hyperactivity (Searle et al., 2013). An RCT study also confirmed the effectiveness of intervention targeted at teacher–student interactions on children’s hyperactive behaviors (Vancraeyveldt et al., 2015). These findings do support initiatives to train teachers in skills that will help them to reduce conflict and promote closeness with their students. Such interventions may be beneficial to the prevention of the development of children’s hyperactivity development.

As far as we know, very few studies have concurrently examined the nature of the influence of maternal TDP, paternal TDP, and teacher’s TDP on children’s hyperactive behaviors; this study was the first one to explore this issue. Consistent with our hypotheses, compared to children with a non-TDP mother, non-TDP father, and non-TDP teacher, those having one parent with TDP, those having both a TDP father and a TDP mother, those having a TDP mother and a TDP teacher, and those having a TDP mother, a TDP father, and a TDP teacher had higher risk of subsequent hyperactive behaviors. The absolute OR was higher among the children with two or more TDP caregivers compared with those with just one TDP caregiver. This indicates the presence of a dose effect. Although previous findings have indicated the association between parental TDP and children’s hyperactive behaviors (He et al., 2019), the present study additionally considered and found the influence of combination effect of maternal TDP, paternal TDP, and teacher’s TDP. These findings support Bronfenbrenner’s bioecological model showing the potential interaction effect between parent factors and teacher factors (Bronfenbrenner and Ceci, 1994). Moreover, some previous studies have given hints to these combination effects. For example, a randomized control trial found that ADHD children whose parents and teacher both took part in the intervention experienced a stronger reduction in hyperactive behaviors than in ADHD children whose parents (but not teachers) took part in the program (Corkum et al., 2005). Another randomized control trial also reported similar results of a greater reduction in children’s ADHD symptoms when both parents and teachers receive the intervention (Ostberg and Rydell, 2012). Such findings indicated that maternal TDP, paternal TDP and teachers’ TDP might combine together and influence the children’s hyperactive behaviors, as demonstrated by our study. This also support a dosage effect, in that being exposed to a teacher with TDP by itself is not sufficient to cause an effect, but when combined with exposure to a mother with TDP, then the combined effect is stronger than exposure to the mother alone.

In addition, our results showed that the children with a TDP father and a TDP teacher, and the children with a TDP mother, a TDP father, and a TDP teacher (baseline hyperactive children) did not show higher risk to have hyperactive behaviors. These results did not illustrate the combination effect of maternal TDP, paternal TDP, and teacher’s TDP, which was insignificant. Though our sample size was relatively large, the number of cases in these two subgroups was no more than 12 cases, which may lead to insufficient statistical power and prevent us to achieve significant results.

### Limitation and Future Research

While this study has some strengths such as being a prospective study with a very large sample size of over 29,000 participants, several limitations ought to also be considered. First, children’s hyperactive behaviors were measured by parental report on the Conners’ HI rather than clinical diagnosis. This was due to the infeasibility to diagnosis by interviews or have direct observations in such a large-scale survey. Instead, the current study used the Conners’ HI, which has been widely employed among preschoolers in epidemiologic research with good validity (Heinonen et al., 2010; Zhu et al., 2015). Second, the current study only measured the personality construct of TDP and so it is not possible to generalize these findings to other personality constructs. Given that there are associations between the Big Five dimensions of personality and TDP (De Fruyt and Denollet, 2002; Denollet, 2005), it would be helpful to replicate this study to include measures of all six dimensions of personality. This will allow the exploration of the unique contributions of each personality dimension toward hyperactive behaviors in children so as to identify the most toxic personality dimensions. Third, we did not collect data on parental mental health other than TDP. Collecting these data would allow a more refined understanding of the relationship between parental TDP and children’s hyperactive behaviors. Fourth, the information of teacher–student relationships was just measured in a subsample of the participants since it is burdensome for the teacher to rate their relationships with each student every year. Fifth, there may exist difference between the respondents and those who did not, so some bias may be introduced. Lastly, our sample was a community sample of preschoolers rather than a clinical sample of older children. Regarding that the problems of hyperactivity cannot be clearly confirmed until older ages when development is stabilized, future researches can be conducted within older children to further confirm the hypotheses.

Given the findings of this study, there are a number of research areas that need further clarification. Since the prevalence of TDP may vary across culture (Bai, 2007; Grande et al., 2010; Martin et al., 2010; Weng et al., 2013), it is important to conduct cross-culture studies to explore the potential influence of culture on these associations. In addition, these findings underscored the importance of examining the effect of the combination of maternal TDP, paternal TDP and teacher’s TDP. This emphasizes the importance of future studies collecting not only the familial information but also the school factors to explore their combined effects on children’s neurobehavioral development. Moreover, the results of this study indicate a need for screening of TDP in parents rather than teachers. Associated with this, the findings of the study suggest that there may be a need to offer specific guidance (e.g., promoting positive rearing behaviors) to TDP parents. However, consistent with a previous study (Vancraeyveldt et al., 2015), our findings do support intervention targeted at establishing positive teacher–student interaction, which may be beneficial to the prevention of the development of children’s hyperactive behaviors. Furthermore, future studies can explore the influence of more other specific variables of teacher–student interaction on children’s hyperactive behaviors, which may help to guide the development of effective intervention to manage hyperactive behaviors of preschoolers.

## Conclusion

This is the first study to find evidence for the effect of combination of teacher’s TDP, maternal TDP, and paternal TDP on the subsequent hyperactive behaviors in young Chinese children. Based on these findings, researchers may consider the effect of combination of teacher’s TDP, maternal TDP, and paternal TDP on hyperactive behaviors in children in further studies.

## Data Availability Statement

The datasets for this manuscript are not publicly available because the datasets are the intellectual and labor property of Sun Yat-sen University and the Longhua Maternal and Child Health Care Center, which cannot be accessed by the public without the permission of these two institutions. Request to access the dataset should be directed to corresponding author.

## Ethics Statement

The studies involving human participants were reviewed and approved by the Ethics Committee of School of Public Health of Sun Yat-sen University. Written informed consent to participate in this study was provided by the participants’ legal guardian/next of kin.

## Author Contributions

W-QC, C-AW, LL, G-HH, Z-LR, HJ, JJ, YJ, and G-MW started and designed the study. W-QC took charge of research training. C-AW took charge of the field investigation. LL, G-HH, Z-LR, X-NY, D-LS, and D-XX participated in the field investigation. G-HH, W-QC, and ES conducted the data analysis and interpretation. G-HH, ES, and W-QC wrote the manuscript. ES revised the manuscript with advice from all authors.

## Conflict of Interest

The authors declare that the research was conducted in the absence of any commercial or financial relationships that could be construed as a potential conflict of interest.

## References

[B1] American Psychiatric Association, (2013). *Diagnostic and Statistical Manual of Mental Disorders Fifth Edition.* Washington, DC: American Psychiatric Publishing.

[B2] Bai J. (2007). Reliability and validity of the Type D personality scale in Chinese. *Chin. Ment. Health J.* 21 329–332. 10.3321/j.issn:1000-6729.2007.05.015

[B3] Barkley R. A. (2014). *Attention-Deficit Hyperactivity Disorder: A Handbook for Diagnosis and Treatment.* New York, NJ: Guilford Publications.

[B4] Baron R. M. Kenny D. A. (1986). The moderator mediator variable distinction in social psychological-research-conceptual, strategic, and statistical considerations. *J. Personal. Soc. Psychol.* 51 1173–1182. 10.1037/0022-3514.51.6.1173 3806354

[B5] Bronfenbrenner U. Ceci S. J. (1994). Nature-nurture reconceptualized in developmental perspective-a bioecological model. *Psychol. Rev.* 101 568–586. 10.1037/0033-295x.101.4.568 7984707

[B6] Buttner S. Pijl S. J. Bijstra J. van den Bosch E. (2015). Personality traits of expert teachers of students with behavioural problems: a review and classification of the literature. *Aust. Educ. Res.* 42 461–481. 10.1007/s13384-015-0176-1

[B7] Chen J. Liu Y. Cai Q. Q. Liu Y. M. Wang T. Zhang K. (2015). Type D personality parents of children with leukemia tend to experience anxiety the mediating effects of social support and coping style. *Medicine* 94:e627. 10.1097/md.0000000000000627 25761192 PMC4602458

[B8] Chorozoglou M. Smith E. Koerting J. Thompson M. J. Sayal K. Sonuga-Barke E. J. (2015). Preschool hyperactivity is associated with long-term economic burden: evidence from a longitudinal health economic analysis of costs incurred across childhood, adolescence and young adulthood. *J. Child Psychol. Psychiatry* 56 966–975. 10.1111/jcpp.12437 26072954 PMC4744758

[B9] Corkum P. V. McKinnon M. M. Mullane J. C. (2005). The effect of involving classroom teachers in a parent training program for families of children with ADHD. *Child Fam. Behav. Ther.* 27 29–49. 10.1300/J019v27n04_02

[B10] De Fruyt F. Denollet J. (2002). Type D personality: a five-factor model perspective. *Psychol. Health* 17 671–683. 10.1080/08870440290025858 23772232

[B11] de Jong R. Mainhard T. van Tartwijk J. Veldman I. Verloop N. Wubbels T. (2014). How pre-service teachers’ personality traits, self-efficacy, and discipline strategies contribute to the teacher-student relationship. *Br. J. Educ. Psychol.* 84 (Pt 2), 294–310. 10.1111/bjep.12025 24829122

[B12] Denollet J. (2005). DS14: standard assessment of negative affectivity, social inhibition, and Type D personality. *Psychosom. Med.* 67 89–97. 10.1097/01.psy.0000149256.81953.49 15673629

[B13] Doumen S. Verschueren K. Buyse E. De Munter S. Max K. Moens L. (2009). Further examination of the convergent and discriminant validity of the student-teacher relationship scale. *Infant Child Dev.* 18 502–520. 10.1002/icd.635

[B14] Eryilmaz A. (2014). Perceived personality traits and types of teachers and their relationship to the subjective well-being and academic achievements of adolescents. *Kuram Ve Uygulamada Egitim Bilimleri* 14 2049–2062. 10.12738/estp.2014.6.2187

[B15] Fletcher J. M. (2014). The effects of childhood ADHD on adult labor market outcomes. *Health Econ.* 23 159–181. 10.1002/hec.2907 23427026 PMC6714576

[B16] Fornell C. Larcker D. F. (1981). Evaluating structural equation models with unobservable variables and measurement error. *J. Mark. Res.* 18 39–50. 10.2307/3151312

[B17] Genc L. Pekic J. Genc A. (2014). The structure of personality of a good teacher from students perspective according to the Big-Five model. *Psihologija* 47 49–63. 10.2298/psi1401049g

[B18] Gillberg C. (2010). The ESSENCE in child psychiatry: early symptomatic syndromes eliciting neurodevelopmental clinical examinations. *Res. Dev. Disabil.* 31 1543–1551. 10.1016/j.ridd.2010.06.002 20634041

[B19] Goyette C. H. Conners C. K. Ulrich R. F. (1978). Normative data on revised conners parent and teacher rating-scales. *J. Abnorm. Child Psychol.* 6 221–236. 10.1007/bf00919127 670589

[B20] Grande G. Romppel M. Glaesmer H. Petrowski K. Herrmann-Lingen C. (2010). The type-D scale (DS14) - norms and prevalence of type-D personality in a population-based representative sample in Germany. *Personal. Individ. Dif.* 48 935–939. 10.1016/j.paid.2010.02.026

[B21] He G. H. Liu L. Strodl E. Ruan Z. L. Jiang H. Jing J. (2019). Parental type D personality and children’s hyperactive behaviors: the mediating role of parent-child interactive activities. *Int. J. Environ. Res. Pub. Health* 16:E116. 10.3390/ijerph16071116 30925765 PMC6480101

[B22] Heinonen K. Raikkonen K. Pesonen A. K. Andersson S. Kajantie E. Eriksson J. G. (2010). Behavioural symptoms of attention deficit/hyperactivity disorder in preterm and term children born small and appropriate for gestational age: a longitudinal study. *BMC Pediatr.* 10:91. 10.1186/1471-2431-10-91 21159164 PMC3012665

[B23] Huang K. L. Wei H. T. Hsu J. W. Bai Y. M. Su T. P. Li C. T. (2018). Risk of suicide attempts in adolescents and young adults with attention-deficit hyperactivity disorder: a nationwide longitudinal study. *Br. J. Psychiatry* 212 234–238. 10.1192/bjp.2018.8 29501070

[B24] Hurtig T. Ebeling H. Jokelainen J. Koivumaa-Honkanen H. Taanila A. (2016). The association between hospital-treated injuries and ADHD symptoms in childhood and adolescence: a follow-up study in the northern Finland birth cohort 1986. *J. Atten. Disord.* 20 3–10. 10.1177/1087054713486699 23665592

[B25] Jingjing Z. Yan L. Yun Z. Coplan R. J. Tiantian L. (2018). Shyness and social adjustment among Chinese preschoolers: the moderating role of student-teacher relationship. *J. Psychol. Sci.* 41 1130–1137. 10.16719/j.cnki.1671-6981.20180516

[B26] Keown L. J. (2012). Predictors of boys’ ADHD symptoms from early to middle childhood: the role of father-child and mother-child interactions. *J. Abnorm. Child Psychol.* 40 569–581. 10.1007/s10802-011-9586-3 22038253

[B27] Keown L. J. Woodward L. J. (2002). Early parent-child relations and family functioning of preschool boys with pervasive hyperactivity. *J. Abnorm. Child Psychol.* 30 541–553. 10.1023/A:1020803412247 12481970

[B28] Lei H. Cui Y. H. Chiu M. M. (2016). Affective teacher student relationships and students’ externalizing behavior problems: a meta-analysis. *Front. Psychol.* 7:1311. 10.3389/fpsyg.2016.01311 27625624 PMC5003892

[B29] Lin-Yan S. Xue-Rong L. Chun Xiang H. Xue Rong L. Zhang J. S. (2001). Norms of the conners parent symptom questionnaire in Chinese urban children. *Chin. J. Clin. Psychol.* 9 241–243. 10.3969/j.issn.1005-3611.2001.04.001 22099201

[B30] Lizhu Y. Miao L. Jinghan C. Yue S. (2016). The influence of teacher expectations on young children’s personality: mediating effect of teacher-children relationship. *Psychol. Dev. Educ.* 32 641–648. 10.16187/j.cnki.issn1001-4918.2016.06.01

[B31] Martin L. A. Doster J. A. Critelli J. W. Lambert P. L. Purdum M. Powers C. (2010). Ethnicity and type D personality as predictors of heart rate variability. *Int. J. Psychophysiol.* 76 118–121. 10.1016/j.ijpsycho.2010.03.001 20211208

[B32] Mols F. Denollet J. (2010). Type D personality in the general population: a systematic review of health status, mechanisms of disease, and work-related problems. *Health Qual. Life Outcomes* 8:9. 10.1186/1477-7525-8-9 20096129 PMC2822747

[B33] O’Connell A. A. Goldstein J. Rogers H. J. Peng C. J. (2008). “Multilevel logistic models for dichotomous and ordinal data,” in *Multilevel Modeling of Educational Data*, eds O’Connell A. A. McCoach D. B. (Charlotte, NC: Information Age Publishing), 199–242.

[B34] Ostberg M. Rydell A. M. (2012). An efficacy study of a combined parent and teacher management training programme for children with ADHD. *Nord. J. Psychiatry* 66 123–130. 10.3109/08039488.2011.641587 22150634 PMC3358125

[B35] Pianta R. C. (2001). *STRS Student-Teacher Relationship Scale-Short Form.* Lutz, FL: Psychological Assessment Resources.

[B36] Prinzie P. Onghena P. Hellinckx W. Grietens H. Ghesquiere P. Colpin H. (2004). Parent and child personality characteristics as predictors of negative discipline and externalizing problem behaviour in children. *Eur. J. Personal.* 18 73–102. 10.1002/per.501

[B37] Runions K. C. (2014). Reactive aggression and peer victimization from pre-kindergarten to first grade: accounting for hyperactivity and teacher-child conflict. *Br. J. Educ. Psychol.* 84 (Pt 4), 537–555. 10.1111/bjep.12037 24796699

[B38] Searle A. K. Miller-Lewis L. R. Sawyer M. G. Baghurst P. A. (2013). Predictors of children’s kindergarten classroom engagement: preschool adult-child relationships, self-concept, and hyperactivity/inattention. *Early Educ. Dev.* 24 1112–1136. 10.1080/10409289.2013.764223

[B39] Shao Y. C. Yin H. L. Wan C. S. (2017). Type D personality as a predictor of self-efficacy and social support in patients with type 2 diabetes mellitus. *Neuropsychiatr. Dis. Treat.* 13 855–861. 10.2147/ndt.S128432 28360523 PMC5365332

[B40] Taylor H. G. Orchinik L. Fristad M. A. Minich N. Klein N. Espy K. A. (2019). Associations of attention deficit hyperactivity disorder (ADHD) at school entry with early academic progress in children born prematurely and full term controls. *Learn. Individ. Dif.* 69 1–10. 10.1016/j.lindif.2018.10.008 31223221 PMC6586420

[B41] Thapar A. Cooper M. Jefferies R. Stergiakouli E. (2012). What causes attention deficit hyperactivity disorder? *Arch. Dis. Child.* 97 260–265. 10.1136/archdischild-2011-300482 21903599 PMC3927422

[B42] Thomas G. de Jong F. Kooijman P. G. C. Cremers C. (2006). Utility of the type D scale 16 and voice handicap index to assist voice care in student teachers and teachers. *Folia Phoniatrica Et Logopaedica* 58 250–263. 10.1159/000093182 16825778

[B43] Vancraeyveldt C. Verschueren K. Wouters S. Van Craeyevelt S. Van den Noortgate W. Colpin H. (2015). Improving teacher-child relationship quality and teacher-rated behavioral adjustment amongst externalizing preschoolers: effects of a two-component intervention. *J. Abnorm. Child Psychol.* 43 243–257. 10.1007/s10802-014-9892-7 25028283

[B44] Weng C. Y. Denollet J. Lin C. L. Lin T. K. Wang W. C. Lin J. J. (2013). The validity of the type D construct and its assessment in Taiwan. *BMC Psychiatry* 13:46. 10.1186/1471-244x-13-46 23379902 PMC3598734

[B45] Williams L. Abbott C. Kerr R. (2016). Health behaviour mediates the relationship between Type D personality and subjective health in the general population. *J. Health Psychol.* 21 2148–2155. 10.1177/1359105315571977 25712490

[B46] Yang C. Bear G. G. Chen F. F. Zhang W. Blank J. C. Huang X. (2013). Students’ perceptions of school climate in the U.S. and China. *Sch. Psychol. Q.* 28 7–24. 10.1037/spq0000002 23506022

[B47] Zhang X. (2010). Reliability and validity of teacher-child relationship scale. *Chin. J. Clin. Psychol.* 18 582–583.

[B48] Zhang Y. F. Sun G. X. (2015). [A Meta analysis of family risk factors for attention deficit hyperactivity disorder]. *Zhongguo Dang Dai Er Ke Za Zhi* 17 721–725. 10.7499/j.issn.1008-8830.2015.07.016 26182279

[B49] Zhu P. Hao J. H. Tao R. X. Huang K. Jiang X. M. Zhu Y. D. (2015). Sex-specific and time-dependent effects of prenatal stress on the early behavioral symptoms of ADHD: a longitudinal study in China. *Eur. Child Adolesc. Psychiatr.* 24 1139–1147. 10.1007/s00787-015-0701-9 25791080

